# Axon growth inhibition by RhoA/ROCK in the central nervous system

**DOI:** 10.3389/fnins.2014.00338

**Published:** 2014-10-22

**Authors:** Yuki Fujita, Toshihide Yamashita

**Affiliations:** ^1^Department of Molecular Neuroscience, Graduate School of Medicine, Osaka UniversityOsaka, Japan; ^2^Japan Science and Technology Agency, Core Research for Evolutional Science and TechnologyTokyo, Japan

**Keywords:** RhoA, ROCK, myelin, axon regeneration, central nervous system

## Abstract

Rho kinase (ROCK) is a serine/threonine kinase and a downstream target of the small GTPase Rho. The RhoA/ROCK pathway is associated with various neuronal functions such as migration, dendrite development, and axonal extension. Evidence from animal studies reveals that RhoA/ROCK signaling is involved in various central nervous system (CNS) diseases, including optic nerve and spinal cord injuries, stroke, and neurodegenerative diseases. Given that RhoA/ROCK plays a critical role in the pathophysiology of CNS diseases, the development of therapeutic agents targeting this pathway is expected to contribute to the treatment of CNS diseases. The RhoA/ROCK pathway mediates the effects of myelin-associated axon growth inhibitors—Nogo, myelin-associated glycoprotein (MAG), oligodendrocyte-myelin glycoprotein (OMgp), and repulsive guidance molecule (RGM). Blocking RhoA/ROCK signaling can reverse the inhibitory effects of these molecules on axon outgrowth, and promotes axonal sprouting and functional recovery in animal models of CNS injury. To date, several RhoA/ROCK inhibitors have been under development or in clinical trials as therapeutic agents for neurological disorders. In this review, we focus on the RhoA/ROCK signaling pathway in neurological disorders. We also discuss the potential therapeutic approaches of RhoA/ROCK inhibitors for various neurological disorders.

## Introduction

Because of the complexity of the CNS and its limited capacity to regenerate on its own, the CNS is one of the most difficult organs to repair after an injury. Various extrinsic and intrinsic factors modulate the onset, severity, and progression of CNS diseases (Yiu and He, 2006). Therapeutic benefits seem to be achieved for diverse CNS diseases by interfering with common targets or pathways. It is evident that lesions to the adult CNS in animals activates RhoA/ROCK signaling pathway and this pathway inhibits axon growth and sprouting. Therefore, this pathway is considered to be a potential therapeutic target for CNS diseases.

Rho family proteins are important regulators of actin dynamics, and regulate cellular shape and motility (Jaffe and Hall, 2005). Multiple extrinsic factors, including myelin-associated inhibitors and RGM, induce the activation of guanine nucleotide exchange factors (GEFs), which promote the exchange of guanosine diphosphate (GDP) for guanosine triphosphate (GTP) binding, thus activating RhoA (Filbin, 2003; Yiu and He, 2003; He and Koprivica, 2004). The GTP-bound form of RhoA causes ROCK activation. RhoA/ROCK activation, in turn, activates downstream effectors, which regulate cytoskeletal reorganization such as growth cone collapse and neurite outgrowth inhibition (Lehmann et al., 1999). Several studies have demonstrated the involvement of the RhoA/ROCK pathway in the pathophysiology of neurological disorders such as spinal cord injury (SCI), optic nerve injury, stroke, and inflammatory CNS diseases (Mueller et al., 2005; Yiu and He, 2006).

After injury, the adult mammalian CNS shows limited regrowth capacity. It has been considered that multiple factors, including the weakness of intrinsic growth capacity, the inhibitory extrinsic environment, and neuronal vulnerability after lesion, cause regenerative failure in the adult CNS. In contrast, the axons in the peripheral nervous system (PNS) or embryonic nervous system show the capacity to regenerate. The difference in the surrounding environment between the CNS and PNS seems to be one of the major causes of limited regeneration of injured CNS axons. In the CNS, axons are ensheathed by myelin formed by oligodendrocytes. When myelinated fibers are damaged after injury, injured CNS axons are exposed to myelin debris that contains inhibitory molecules for axonal regrowth. These inhibitory molecules induce the activation of RhoA/ROCK in neurons. In addition, the developmental guidance molecules such as ephrin B3 and semaphorin 4D (Sema4D/CD100) are also expressed in CNS myelin, suggesting that they are involved in the inhibitory effect of CNS myelin (Moreau-Fauvarque et al., 2003; Benson et al., 2005). Further, the glial scar formed by reactive astrocytes also inhibits axonal regrowth by releasing axonal inhibitory molecules such as chondroitin sulfate proteoglycans (CSPGs). CSPGs also trigger the activation of RhoA/ROCK, resulting in outgrowth inhibition. Thus, multiple axon growth inhibitory molecules converge on RhoA/ROCK in neurons. In this section, we summarize the molecular signals mediated by axon growth inhibitors. There are at least two members of the ROCK family, ROCK-I and ROCK-II (Nakagawa et al., 1996). Both of them contain an amino-terminal kinase domain, a Rho-binding domain (RBD), which is located within the mid-coiled-coil-forming domain, and a pleckstrin-homology (PH) domain with a carboxy-terminal cysteine-rich domain (CRD) (Riento and Ridley, 2003; Mueller et al., 2005). ROCK-II is mainly expressed in brain and skeletal muscle, whereas ROCK-I is prominently expressed in non-neuronal tissues such as the liver, testis, and kidney (Nakagawa et al., 1996). Although PNS neurons are also myelinated by Schwann cells, myelin debris in the PNS seems to be removed much faster and more effectively compared to the CNS (Griffin et al., 1992; George and Griffin, 1994). Further, some of myelin-associated inhibitors such as Nogo are expressed only in CNS myelin but not PNS myelin (Yiu and He, 2006). This review summarizes the molecular mechanisms of CNS disorders mediated by RhoA/ROCK signaling. We also discuss the potential use of RhoA/ROCK inhibitors as a therapeutic strategy to treat CNS disorders.

## RhoA/ROCK signaling and axon growth inhibition

### Myelin-associated inhibitors

Three myelin-associated inhibitors—Nogo, MAG, and oligodendrocyte-myelin glycoprotein (OMgp)—have been well characterized. Nogo was identified (Chen et al., 2000; Grandpre et al., 2000; Prinjha et al., 2000) as the putative antigen of the monoclonal antibody IN-1 (Caroni and Schwab, 1988; Schnell and Schwab, 1990), and neutralized the inhibitory effects of myelin. Nogo has at least three different isoforms generated by alternative splicing and promoter usage (Nogo-A, Nogo-B, and Nogo-C). Nogo-A is mainly expressed in the nervous system; Nogo-A is found in the endoplasmic reticulum, and also is observed at lower levels on the cell surface of the myelin sheath (Grandpre et al., 2000; Wang et al., 2002c). In contrast, Nogo-B and Nogo-C are widely expressed outside the CNS (Huber et al., 2002). Nogo-A/B expression is not altered significantly after spinal cord injury (Huber et al., 2002). Nogo has two transmembrane domains, and these domains are separated by a 66-amino acid loop, Nogo-66, which is shared by all three isoforms. Nogo-66 is one of the inhibitory domains of Nogo and causes growth cone collapse (Fournier et al., 2001).

MAG was the first identified potent inhibitor of neurite outgrowth (McKerracher et al., 1994; Mukhopadhyay et al., 1994). It is a transmembrane protein with five immunoglobulin-like domains in its extracellular region, and is localized in both PNS Schwann cells and CNS oligodendrocytes of myelin sheaths. MAG is required for the formation and maintenance of myelin (Quarles, 2007). MAG has bidirectional effects on axonal growth; in young neurons, MAG promotes axonal growth, whereas in older neurons, it inhibits axonal growth (Johnson et al., 1989; Salzer et al., 1990; McKerracher et al., 1994; Mukhopadhyay et al., 1994; Debellard et al., 1996; Turnley and Bartlett, 1998). This bidirectional effect of MAG on neurons seems to depend on intracellular levels of cyclic AMP (cAMP). The endogenous cAMP level is higher in young neurons than in older neurons, thus converting MAG from a promoter to an inhibitor of axonal growth (Cai et al., 2001). Further, some studies reported the protective effects of MAG on the neurons (Nguyen et al., 2009; Lee et al., 2010; Kinter et al., 2012; Jones et al., 2013). Deletion of MAG reduces corticospinal tract (CST) sprouting after pyramidotomy *in vivo* (Lee et al., 2010). MAG prevents vincristine-induced axonal degeneration in postnatal dorsal root ganglion neurons (Nguyen et al., 2009). Thus, MAG has both inhibitory and promoting effects on axonal growth in mature neurons.

OMgp is a glycosylphosphatidylinositol (GPI)-anchored glycoprotein with a leucine-rich repeat (LRR) domain (Kottis et al., 2002; Wang et al., 2002b). OMgp is expressed in both oligodendrocytes and neurons (Habib et al., 1998). During development, OMgp-null mice show impaired myelination and thalamo-cortical projection (Gil et al., 2010; Lee et al., 2011). Although deletion of OMgp does not improve axon regeneration after SCI (Ji et al., 2008; Cafferty et al., 2010; Lee et al., 2010), its removal promotes sprouting of serotonergic axons (Ji et al., 2008). The highest level of OMgp mRNA at the lesion site is detected 1 day after SCI (Guo et al., 2007).

**Figure 1:**
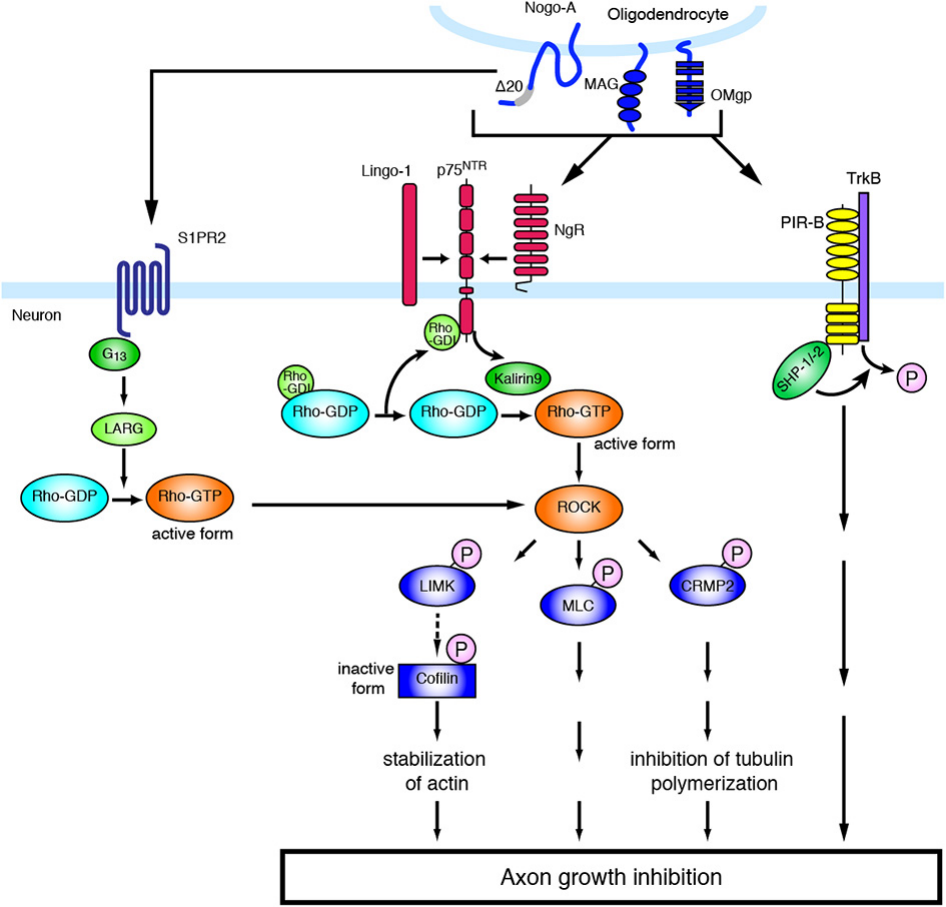
**Molecular mechanisms of inhibitory environmental molecules in axon growth inhibition**. The adult mammalian CNS shows limited capacity for axon regeneration. Myelin-associated inhibitors such as MAG, Nogo, and OMgp bind to NgR1 and PIR-B, whereas Nogo-A-Δ-20 specifically binds to S1PR2. Myelin-associated inhibitors transduce signals to neurons through NgR, which is part of a receptor complex, including p75^NTR^ and Lingo-1. The ligand binding to NgR induces the activation of RhoA/ROCK. The activation of ROCK leads to the phosphorylation of various substrates, resulting in axon growth inhibition.

**Figure 2:**
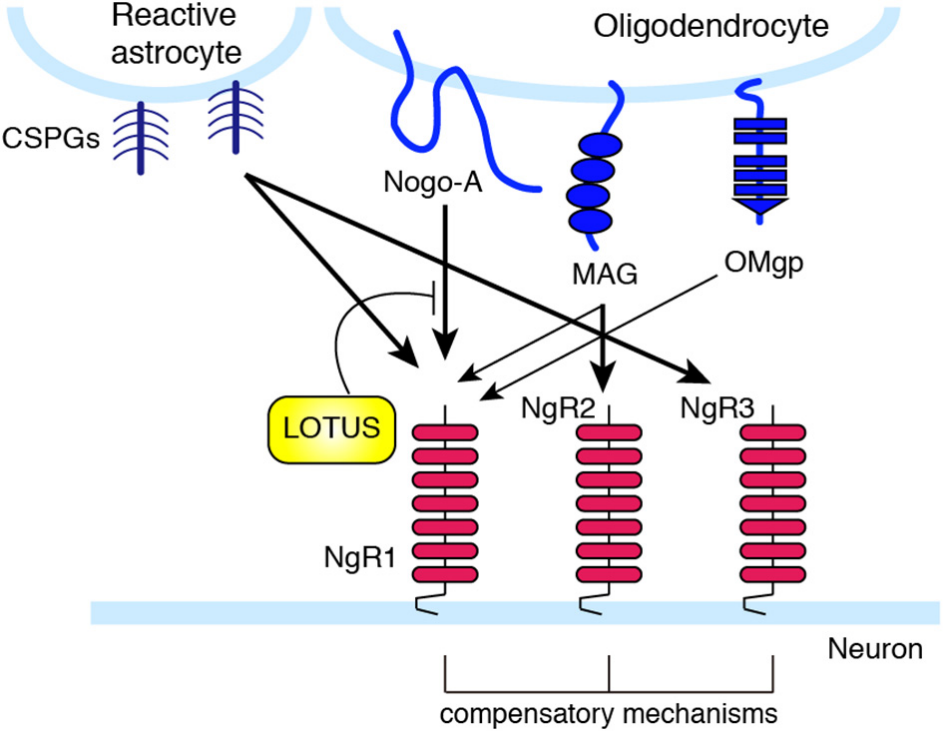
**Nogo receptor family members and their ligand selectivity**. NgR1 interacts with MAG, Nogo, and OMgp. NgR2 binds to MAG with high affinity, and has redundant function to NgR1 in MAG-induced neurite outgrowth inhibition. LOTUS interacts with NgR1, and inhibits the binding of Nogo to NgR. CSPGs bind with high affinity to NgR1 and NgR3.

These three structurally distinct proteins all bind to the same receptor, the Nogo receptor (NgR) (Fournier et al., 2001; Domeniconi et al., 2002; Liu et al., 2002; Wang et al., 2002b) and the paired immunoglobulin-like receptor B (PIR-B) (Atwal et al., 2008) (Figure 1). Among the NgR family receptor (NgR1, NgR2, and NgR3), NgR1 was first identified. Later, NgR2 and NgR3 were discovered as proteins bearing sequence similarities to NgR1 (Barton et al., 2003; Lauren et al., 2003; Pignot et al., 2003) (Figure 2). MAG can bind to NgR2 with higher affinity than to NgR1 (Venkatesh et al., 2005). Deletion of either NgR1 or NgR2 does not affect the MAG-mediated neurite growth inhibition in sensory neurons (Worter et al., 2009). NgR1 and NgR3 bind to CSPG, and mediate the inhibitory effect of CSPG in cultured neurons (Dickendesher et al., 2012). Knockdown of NgR1 along with NgR3, but not single knockdown of either receptor, promotes axonal regeneration after optic nerve injury. These observations suggest that there are redundant and compensatory mechanisms among these receptors.

Since NgR is a GPI-anchored protein and has no intracellular domain, NgR is considered unable to transduce signals into neurons and requires a co-receptor(s). The low-affinity neurotrophin receptor p75^NTR^ was found to be a signal transducer of MAG (Yamashita et al., 2002), and subsequent studies demonstrated that p75^NTR^ associates with NgR to form a receptor complex for MAG, Nogo, and OMgp (Wong et al., 2002; Wang et al., 2002a). The CNS transmembrane protein leucine-rich repeat and Ig domain containing 1 (Lingo-1) was also identified as an additional component of the receptor complex of NgR and p75^NTR^ (Mi et al., 2004). p75^NTR^ induces the release of RhoA from Rho GDP-dissociation inhibitor (RhoGDI), thus acting as a RhoGDI dissociator (Yamashita and Tohyama, 2003). In addition, the RhoGEF Kalirin9 directly binds to p75^NTR^, and competes with RhoGDI for binding to p75^NTR^. MAG reduces the interaction of Kalirin9 with p75^NTR^, resulting in the increased association of RhoGDI to p75^NTR^ (Harrington et al., 2008). This causes the activation of RhoA/ROCK signaling, leading to growth cone collapse and axon growth inhibition. Indeed, the ROCK inhibitor Y-27632 attenuates the inhibitory effect of these myelin-associated inhibitors. Lingo-1 seems to also regulate the localization of RhoGDI and the activation of RhoA (Zhang et al., 2009). Downstream of the RhoA/ROCK signaling pathway, inactivation of collapsin response mediator protein-2 (CRMP-2) inhibits neurite outgrowth. CRMP-2 interacts with tubulin heterodimers and facilitates microtubule assembly (Fukata et al., 2002). Overexpression of CRMP-2 promotes axonal growth (Inagaki et al., 2001). Upon MAG stimulation in cultured cerebellar neurons, CRMP-2 is phosphorylated and inactivated by ROCK (Mimura et al., 2006). These observations suggest that the inactivation of CRMP-2 by ROCK-mediated phosphorylation inhibits tubulin polymerization, leading to neurite outgrowth inhibition. In addition, it is demonstrated that RhoA/ROCK pathway also regulates actin cytoskeleton (Ohashi et al., 2000; Sumi et al., 2001; Hsieh et al., 2006). Ser/Thr kinase, LIM (Lin-11, Isl-1, and Mec-3) kinase is phosphorylated by ROCK, leading to the phosphorylation of actin depolymerization factor, cofilin. Inactivation of cofilin by LIM kinase stabilizes the actin filament in the growth cone, resulting in the inhibition of neurite outgrowth. Recently, cartilage acidic protein-1b (lotus) was identified as a protein that endogenously antagonizes NgR1 (Sato et al., 2011; Kurihara et al., 2012). Lotus suppresses Nogo-NgR1 binding and Nogo-induced growth cone collapse. Interestingly, lotus-deficient mice show defasciculated lateral olfactory tracts, suggesting that lotus is required for normal fasciculation of the specific tract in the CNS.

NgR is not to be an only receptor to mediate neurite outgrowth inhibition. Previous studies reported that genetic deletion of NgR does not reduce neurite outgrowth inhibition by myelin-derived proteins *in vitro*, nor does it enhance axon regeneration after SCI (Kim et al., 2004; Zheng et al., 2005; Chivatakarn et al., 2007). These observations suggest that unidentified receptors may be involved in the neurite outgrowth inhibition induced by myelin-derived axon growth inhibitors. Indeed, PIR-B, which is a major histocompatibility complex (MHC) class I receptor (Takai, 2005), was identified as the second receptor that inhibits neurite extension (Atwal et al., 2008).

The above-mentioned molecules have some functions also in the physiological conditions. Several reports demonstrate that NgR signaling limits synaptic plasticity. In the visual cortex, deletion of NgR delayed the closure of critical period for ocular dominance plasticity to monocular deprivation (McGee et al., 2005). NgR1 regulates dendritic spine morphology and activity-dependent synaptic plasticity (Lee et al., 2008). Further, postsynaptic NgR1 limits synapse formation in the hippocampus during CNS development through the activation of RhoA (Wills et al., 2012). These observations indicate that NgR regulates the proper development of the nervous system. NgR expression is decreased specifically in the sensory-deprived cortex and in adjacent region after SCI (Endo et al., 2007). These results suggest the involvement of NgR in cortical plasticity after the injury, and that decreased expression of NgR may facilitate neural rewiring after injury in the CNS. PIR-B was first identified to regulate ocular-dominance plasticity (Syken et al., 2006), indicating that PIR-B restricts neuronal plasticity in physiological conditions. Further, a recent report demonstrates that the G protein-coupled receptor (GPCR) sphingosine-1-phosphate receptor 2 (S1PR2) is a receptor for Nogo-A, and Nogo-A restricts synaptic plasticity through this receptor in physiological conditions (Kempf et al., 2014). Nogo-A-Δ-20 (amino-Nogo), which is the N-terminal extracellular region of Nogo-A, binds to S1PR2 and induces the activation of the G protein G_13_, the RhoGEF LARG, and RhoA. Inhibition of Nogo-A/S1PR2 signaling increases hippocampal and cortical long-term potentiation. Thus, physiological Nogo signaling seems to regulate synaptic plasticity.

### RGMa

Repulsive guidance molecule (RGM) also acts as an inhibitor of axon growth (Mueller et al., 2006; Yamashita et al., 2007). RGM is a GPI-anchored membrane-bound protein with a molecular weight of 33/35 kDa. RGM has been shown to bind to its receptor, neogenin. RGM was originally identified as a protein that induces growth cone collapse in the chick retinotectal system during development. Three homologs of RGM have been identified in mouse and human, including RGMa, RGMb, and RGMc. In the adult nervous system, RGMa has a role in inhibiting axon regeneration. RGMa expression is increased around the lesion after SCI. RGMa-positive microglia/macrophages and oligodendrocytes are observed in the lesion epicenter area. Treatment with neutralizing anti-RGMa antibodies after SCI in rat promotes axonal regeneration and functional recovery (Hata et al., 2006). RGMa binds to its receptor neogenin, and then induces the association of UNC5B and neogenin. LARG, which is a RhoGEF, is recruited to this receptor complex and induces RhoA activation (Hata et al., 2009). A ROCK inhibitor indeed blocks RGMa-induced neurite outgrowth inhibition, demonstrating that RGMa inhibits neurite outgrowth via activation of the RhoA/ROCK pathway. The phosphorylation of myosin light chain (MLC) is increased after SCI. Inhibition of myosin IIA prevents neurite outgrowth inhibition induced by RGMa (Kubo et al., 2008). These results indicate that ROCK induces phosphorylation of myosin light chain (MLC), leading to myosin IIA activation, and that this effect is essential for neurite outgrowth inhibition induced by RGMa. In addition, transcriptional coactivator LIM domain only 4 (LMO4) is also involved in RGMa-induced RhoA activation (Schaffar et al., 2008). LMO4 directly interacts with cytoplasmic domain of neogenin in cortical neurons. Binding RGMa to neogenin leads to the dissociation of LMO4 from neogenin. This dissociation increases the interaction of LMO4 with other molecules, such as Src homology 2-containing protein tyrosine phosphatase (SHP)-2 (Novotny-Diermayr et al., 2005), resulting in RhoA activation. Knockdown of LMO4 prevents RGMa-induced RhoA activation and neurite outgrowth inhibition. Thus, RGMa induces activation of RhoA through the release of LMO4 from neogenin.

### CSPGs

**Figure 3:**
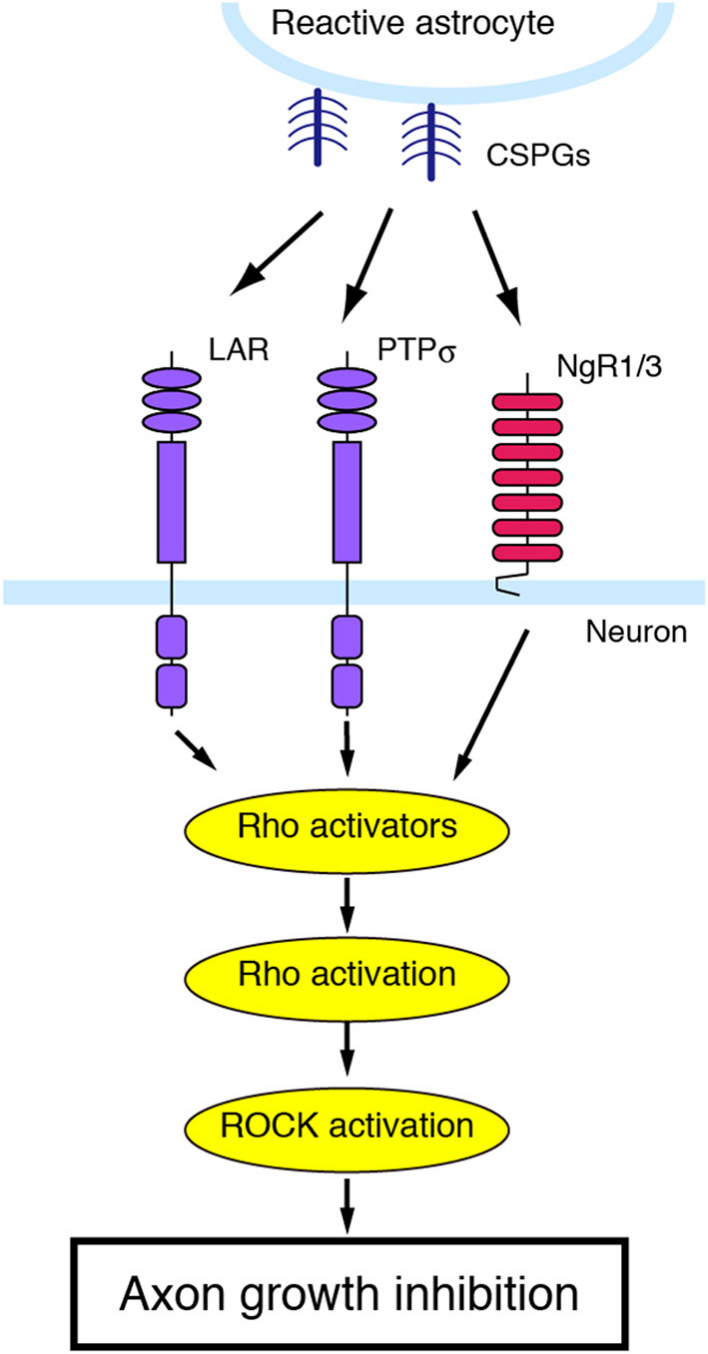
**CSPGs and their receptors**. PTPσ, LAR, which is another member of the LAR subfamily of receptor protein tyrosine phosphatase, and NgR1/3 act as a receptor for CSPGs. Blockade of these receptors can reverse the inhibitory effects of CSPGs on neurite growth.

At the lesion site, the glial scar blocks axon regrowth. The glial scar is mainly formed by reactive astrocytes. Although astrocytes have been shown to generate growth-promoting molecules, they also produce inhibitory extracellular matrix molecules such as proteoglycans. Proteoglycans consist of a core protein attached by sugar moieties to a sulfated glycosaminoglycan chain with disaccharide repeats. Astrocytes produce four types of proteoglycans, based on the composition of the repeating disaccharide: CSPG, dermatan sulfate proteoglycan, keratan sulfate proteoglycan, and heparan sulfate proteoglycan. Among these, CSPGs play important roles to inhibit axonal regeneration after injury. CSPGs include aggrecan, brevican, neurocan, NG2, phosphacan, and versican, all of which have chondroitin sulfate chains. In the adult mammalian CNS, CSPGs are secreted by reactive astrocytes immediately after injury and their expression persists for a long period (McKeon et al., 1999; Jones et al., 2003; Tang et al., 2003). For instance, the expression of neurocan and versican is upregulated for 4 weeks after SCI. A transmembrane protein tyrosine phosphatase, PTPσ, transduces CSPG-mediated inhibition of axon growth (Shen et al., 2009) (Figure 3). CSPGs interact with the first immunoglobulin-like domain of PTPσ. Genetic deletion of PTPσ enhances axon sprouting to the regions containing CSPGs in the animal model of SCI. Leukocyte common antigen-related phosphatase (LAR), which is another member in the LAR subfamily of PTPσ, is also identified as a CSPG receptor (Fisher et al., 2011). After SCI, deletion of LAR reverses CSPG-induced neurite growth inhibition. Blocking LAR with a peptide enhances axonal growth of serotonergic fibers. Further, CSPGs inhibit neurite outgrowth through the activation of the RhoA/ROCK pathway since inhibiting RhoA/ROCK signaling blocks the inhibitory effects of CSPGs on neurite outgrowth (Dergham et al., 2002; Borisoff et al., 2003; Monnier et al., 2003).

## RhoA/ROCK inhibition in animal models of CNS disorders

### Inhibition of RhoA/ROCK signaling in SCI

It is evident that minimal axon regeneration occurs after SCI in adult animals (Schwab and Bartholdi, 1996). Previous studies have addressed whether axon regeneration can be induced by pharmacological treatments or genetic manipulations that target inhibitory axon growth signals. While the treatment of neurons with MAG, Nogo, and OMgp inhibits neurite outgrowth *in vitro*, it is still under debate whether they also inhibit axonal outgrowth in the CNS *in vivo*. Intracerebral or Intrathecal administration of neutralizing antibodies for Nogo (designated as IN-1), implantation of IN-1 mAb-secreting hybridoma cells (Schnell and Schwab, 1990; Bregman et al., 1995; Brosamle et al., 2000; Merkler et al., 2001), or NgR antagonist peptide NEP1-40 (Nogo extracellular peptide, residues 1–40) enhances axonal outgrowth and functional recovery after SCI (Grandpre et al., 2002; Li and Strittmatter, 2003). However, it should be noted that there are conflicting reports about the effects of genetic deletion of Nogo or NgR on CNS injury (Kim et al., 2003, 2004; Simonen et al., 2003; Zheng et al., 2003, 2005; Cafferty and Strittmatter, 2006; Dimou et al., 2006). Deletion of PIR-B, which is another receptor for MAG, Nogo, and OMgp, demonstrates no improvement in axonal regeneration and motor function after SCI (Nakamura et al., 2011). Moreover, a triple-deletion of MAG, Nogo, and OMgp fails to facilitate the regeneration or functional recovery after SCI compared to wild type mice (Lee et al., 2010). These observations suggest that achieving a beneficial outcome by targeting an individual ligand or receptor is difficult, presumably because multiple signals mediate axon growth inhibition.

On the other hand, RhoA/ROCK is the shared signal among multiple inhibitory factors. Therefore, this pathway may be a promising molecular target for the treatment of axon regeneration. It has been reported that RhoA activation is observed in the lesion site after SCI (Dubreuil et al., 2003; Madura et al., 2004). In addition, RhoA inactivation exerts *in vivo* therapeutic effects on SCI. Treating mice with a single injection or gel foam patches application of C3 transferase (C3), which inactivates RhoA by ADP ribosylation of the effector domain, promotes axonal regrowth of CST fibers after SCI, and improves locomotion (Dergham et al., 2002; Boato et al., 2010). Further work shows that a cell-permeable derivative of C3, C3-05, reverses RhoA activation and prevents p75^NTR^-dependent cell death (Dubreuil et al., 2003). Thus, RhoA inhibition mediates axonal regrowth and neuroprotection, leading to functional recovery after SCI. ROCK inhibition also causes beneficial effects on SCI. Intraperitoneal or intrathecal treatment, or gel foam implant of ROCK inhibitors fasudil and Y-27632 stimulate axonal regrowth and functional recovery in a SCI model (Hara et al., 2000; Dergham et al., 2002; Fournier et al., 2003; Sung et al., 2003). Furthermore, the peptide inhibitor of p21^CIP1/WAF1^ (ROCK inhibitory protein) also promotes axonal growth and functional recovery (Tanaka et al., 2004).

### Inhibition of RhoA/ROCK signaling in optic nerve injury

Previously, deleting MAG was reported to have almost no effect on optic nerve regeneration after crush injury (Bartsch et al., 1995). In contrast, inhibition of downstream molecules can provide an alternative approach. Inactivation of RhoA with the treatment of C3 or its derivatives intravitreously, by using gel foam, or by injection of adeno-associated viruses carrying a gene for C3 ribosyltransferase promotes *in vivo* axonal regrowth after optic nerve injury (Lehmann et al., 1999; Fischer et al., 2004; Bertrand et al., 2005). Intravitreous injections of ROCK inhibitors, including Y-27632 and dimethyl-fasudil also improve optic nerve regeneration (Lingor et al., 2007, 2008). These findings indicate that RhoA/ROCK could be a promising target for the treatment of axon degeneration. We have recently demonstrated that inhibition of PIR-B signaling promoted optic nerve regeneration after crush injury (Fujita et al., 2011). Upon MAG stimulation, PIR-B interacts with tropomyosin receptor kinase (Trk) neurotrophin receptors, which are known to promote neurite outgrowth. This receptor complex recruits SHP, leading to dephosphorylation and inactivation of Trk receptors. Inhibition of SHP induces axonal regrowth after optic nerve injury. However, *Pirb*-knockout (KO) mice show little to no detectable regeneration of optic nerve fibers. Intravitreous treatment with brain-derived neurotrophic factor (BDNF), which is a ligand for TrkB, promotes axonal regrowth in *Pirb*-KO mice but not in wild type mice. These results suggest that for axonal regeneration after optic nerve injury, both inhibition of the negative signal and activation of the positive signal on axon growth are required.

### Inhibition of RhoA/ROCK signaling in stroke and traumatic brain injury

The inhibition of the RhoA/ROCK pathway also has neuroprotective effects after stroke. Several studies using rodent stroke models demonstrate that inhibiting the Nogo–NgR pathway promotes functional recovery (Wiessner et al., 2003; Lee et al., 2004). As an experimental stroke model in rodents, middle cerebral artery occlusion (MCAO) is widely used. Mice lacking NgR or Nogo-A/B exhibit improved functional recovery after MCAO (Lee et al., 2004). Intraventricular treatment with a purified monoclonal anti-NogoA antibody (7B12) 24 h after injury promotes sprouting of corticospinal fibers and improves long-term functional recovery (Wiessner et al., 2003). However, controversial results have been reported regarding the effect of PIR-B inhibition on CNS injury. Mice lacking PIR-B show no difference in the sprouting of CST axons and functional recovery compared with wild type mice after traumatic brain injury or SCI (Omoto et al., 2010; Nakamura et al., 2011). In contrast, increased crossing of CST fibers from the intact motor cortex and improved behavioral outcome could be observed after MCAO in *Pirb*-KO mouse (Adelson et al., 2012). These discrepancies might be due to different experimental conditions such as different *Pirb*-KO mice or different disease models.

There is also evidence from the human brain demonstrating the association of the RhoA/ROCK pathway with CNS diseases. It has been reported that RGM expression is increased in lesion and perilesion sites in adult human brains with focal cerebral ischemia or traumatic brain injury (Schwab et al., 2005). Furthermore, the expression of RhoA and B at the lesion site is increased in the autopsied tissue of humans dying from traumatic brain injury (Brabeck et al., 2004). Indeed, mice treated with the RhoA/ROCK inhibitors such as C3, fasudil, hydroxyfasudil, and Y-27632 subcutaneously, intraperitoneally, or intravenously show increased recovery after ischemia-induced damage (Laufs et al., 2000; Toshima et al., 2000; Satoh et al., 2001; Rikitake et al., 2005). These results suggest that reversing the RhoA/ROCK pathway may contribute to restoration of the injured CNS network after stroke.

### Inhibition of RhoA/ROCK signaling in neurodegenerative disorders

Alzheimer's disease (AD) is characterized by pathological markers in the brain such as deposition of the beta-amyloid peptide (Aβ) and intracellular neurofibrillary tangles. Some non-steroidal anti-inflammatory drugs (NSAIDs) have been shown to reduce the risk of AD development (McGeer et al., 1996; Anthony et al., 2000; Weggen et al., 2001; Eriksen et al., 2003). It has been reported that NSAIDs can reduce the Aβ production by blocking the RhoA/ROCK signaling. Inhibition of ROCK by Y-27632 reduced the level of Aβ 1–42, which is more prone to aggregation than Aβ 1–40 in AD model mice (Zhou et al., 2003). In contrast, other studies have demonstrated that the NSAIDs reduce the production of Aβ by the inhibiting of γ-secretase pathway (Takahashi et al., 2003; Beher et al., 2004). Aβ is produced by the cleavage of amyloid precursor protein (APP) by β- and γ-secretases. In contrast, since APP cleavage by α-secretase occurs within the Aβ sequence, the production of Aβ is avoided (Esch et al., 1990; Sisodia et al., 1990). Inhibition of RhoA/ROCK signaling by statins seems to induce APP cleavage by α-secretase, leading to the reduction of Aβ generation (Pedrini et al., 2005). It has been shown that NgR family members associate with APP processing. Subcellular localization of NgR and Nogo is altered in AD brain. Overexpression of NgR decreases Aβ generation, and deletion of NgR in an AD model mouse increases Aβ accumulation (Park et al., 2006). All three NgRs can interact with APP, and the interaction of NgR2 with APP favors processing of APP by β-secretase. Deletion of NgR2 in AD model mouse reduces Aβ deposition (Zhou et al., 2011). These observations suggest that inhibition of RhoA/ROCK signaling shows differential effects on Aβ generation.

Further, involvement of RhoA/ROCK signaling has been suggested in other neurodegenerative diseases, such as Parkinson's disease, Huntington's disease, and amyotrophic lateral sclerosis (ALS). Oral gavage administration of fasudil in 1-methyl-4-phenyl-1,2,3,6-tetrahydropyridine (MPTP) mouse model of Parkinson's disease reduced the loss of dopaminergic neurons. ROCK inhibition induces Akt activation, leading to neuroprotective effect (Tonges et al., 2012). ROCK inhibitor Y-27632 prevents the phosphorylation of profilin, leading to the inhibition of huntingtin aggregation (Shao et al., 2008). ROCK inhibitor and ROCK-II siRNA prevent striatal neuronal death induced by polyQ-Huntingtin *in vitro* (Deyts et al., 2009). In the hSOD1^G93A^ mouse, which is the animal model of ALS, ROCK is upregulated and ROCK-pMLC pathway seems to be involved in the synapse loss (Hu et al., 2003; Sunico et al., 2011). Inactivation of ROCK by oral gavage administration of Y-27632 improves the survival of spinal muscular atrophy (SMA) model mice (Bowerman et al., 2010). These observations suggest that inhibition of Rho/ROCK pathway can be a therapeutic lead for diverse neurodegenerative disorders.

### Immune system-mediated CNS diseases

Multiple sclerosis (MS) is characterized by inflammation, demyelination, and axonal loss. Patients with MS exhibit various neurological signs such as motor deficits, progressive paralysis, and optic neuritis. The immune system plays a key role in the pathogenesis of MS (Noseworthy et al., 2000; Hauser and Oksenberg, 2006; Trapp and Nave, 2008; Krumbholz and Meinl, 2014). However, the precise pathogenesis of MS is unclear. It has been historically considered that a patient's own immune system attacks CNS myelin as foreign and then destroys it; this destructive process induces axonal damage. One possible mechanism in the pathogenesis of MS is the migration of leukocytes into the CNS, which induces inflammatory signals and CNS demyelination. Statins, drugs widely used for lowering cholesterol, are considered to prevent the infiltration of leukocytes into the CNS. Although statins inhibit 3-hydroxy-3-methylglutaryl coenzyme A (HMG-CoA) reductase, they also inhibit isoprenylation of RhoA, which inactivates RhoA (Neuhaus et al., 2004). RhoA inhibition suppresses leukocyte infiltration into CNS in an experimental autoimmune encephalomyelitis (EAE), which is the primary animal model of MS (Walters et al., 2002; Greenwood et al., 2003; Hendriks et al., 2004).

Several studies have demonstrated the role of ROCK in inflammatory disorders such as MS (Sun et al., 2006; Yu et al., 2010; Hou et al., 2012). The ROCK inhibitor fasudil has been shown to inhibit the development of EAE (Sun et al., 2006). Fasudil reduces T-cell proliferation and infiltration of inflammatory cells into the CNS (Yu et al., 2010). The upregulation of ROCK-II in perivascular spaces and vascular endothelial cells of the spleen, spinal cord, and brain in EAE are all inhibited by fasudil treatment. The molecular mechanism underlying the inhibitory effect of fasudil may include regulating cytokine production. Compared with a control group, fasudil treatment in EAE mice downregulates interleukin (IL)-17, IL-6, and monocyte chemoattractant protein-1 (MCP-1), whereas it upregulates IL-4 (Yu et al., 2010). In addition, fasudil also inhibits toll-like receptor-4 (TLR-4), phosphorylation of nuclear factor κ B (NF-κ B)/p65, and inflammatory cytokines such as IL-1β and tumor necrosis factor (TNF)-α, and enhanced IL-10 production in spinal cord (Hou et al., 2012). These observations suggest that inhibition of the RhoA/ROCK pathway may contribute to neuroprotection in MS.

T-cell activation and trafficking within the CNS have been considered to mediate the processes in MS (Barten et al., 2010; Hilas et al., 2010; Chastain et al., 2011). It is well established that antigen-presenting cells (APCs) activate T cells by presenting MHCs to the T-cell receptor (Guermonprez et al., 2002). Activated T cells can transmigrate across the blood-brain barrier (BBB) and locate to the CNS. They are re-stimulated by APCs, which trigger disease progression (Tompkins et al., 2002). For example, suppression of T cells by Fingolimod (FTY720), which is a sphingosine-1-phosphate (S1P) receptor agonist, contributes to attenuation of autoimmune activity in MS (Brown et al., 2007). Thus, suppressing the immune system can help to slow the progression of MS. Our recent study demonstrates that RGMa mediates T-cell activation, and inhibition of RGMa attenuates clinical symptoms of EAE (Muramatsu et al., 2011). We previously found that RGMa was expressed outside the CNS, in particular in the activated macrophages/microglia after SCI (Hata et al., 2006). Further analysis revealed that RGMa was expressed in bone marrow-derived dendritic cells upon stimulation with lipopolysaccharide, and its receptor, neogenin was expressed in CD4^+^ T cells. These observations suggest that RGMa mediates CD4^+^ T-cell activation triggered by dendritic cells. Binding of RGMa to CD4^+^ T cells activates Rap1, leading to stronger adhesion of T cells to intracellular adhesion molecule-1 (ICAM-1). Neutralizing anti-RGMa antibody reduces the infiltration of inflammatory cells into the CNS and blocks clinical signs of EAE. Further, RGMa expression is increased in dendritic cells in the brain and spinal cord of patients with MS. Neutralizing anti-RGMa antibody inhibits the proliferation of peripheral blood mononuclear cells prepared from patients with MS and reduces the production of inflammatory cytokines interferon-γ (IFN-γ), ILs-2, −4, and −17. These results suggest that RGMa is involved in the pathogenesis of MS through the activation of CD4^+^ T cells, although involvement of RhoA/ROCK remains to be determined.

## RhoA/ROCK inhibitors

Since multiple inhibitory signals converge onto the RhoA/ROCK pathway, identifying RhoA/ROCK inhibitors would be helpful in the treatment of neurological disorders. The Rho inhibitor C3 enzyme was originally identified from *Clostridium botulinum* culture supernatants. C3 specifically ADP-ribosylates Rho on its effector domain without inhibiting the activity of other members of the Rho family. To date, seven members of C3-like transferases have been identified. The first evidence for the effect of RhoA inhibition to promote axon regeneration *in vivo* was provided by studies on an optic nerve injury model; treatment with C3 enhances optic nerve regeneration (Lehmann et al., 1999). Thereafter, it was demonstrated that C3 promotes axonal regrowth and functional recovery after SCI (Dergham et al., 2002). Treatment with C3-05, the cell-permeable form of C3, in a fibrin matrix to the lesion site after injury shows ability to suppress RhoA activation to a physiological level (Dubreuil et al., 2003). Administration of BA-210 (Cethrin®), a recombinant fusion protein composed of C3, inactivates RhoA and improves functional recovery after SCI in rodents (Lord-Fontaine et al., 2008). Based on these observations, phase I/II clinical trials of BA-210 were conducted (Fehlings et al., 2011).

Several ROCK inhibitors have also been discovered. Isoquinoline derivatives are typical ROCK inhibitors. Fasudil (hexahydro-1-(5-isoquinolylsulfonyl)-1H-1,4-di-azepime, also known as HA-1077), which has the isoquinoline and the homopiperazine ring, is widely used as a ROCK inhibitor. Although fasudil effectively inhibits ROCK, it also inhibits several protein kinases, including PRK2 and MSK1 (Davies et al., 2000). Hydroxyfasudil is the major metabolite of fasudil *in vivo*. It is slightly more active than the original compound and has a longer half-life [fasudil: *t*_(1/2)_ = 0.3 h, hydroxylfasudil: *t*_(1/2)_ = 2.9 h] (Chen et al., 2010). Although both fasudil and hydroxyfasudil show low infiltration ability into the brain, preparation of either in liposomes can improve their efficacy (Ishida et al., 2006). Another isoquinoline derivative, dimethylfusudil (H-1152P), was optimized on the basis of fasudil and shows higher efficacy and selectivity for ROCK (Sasaki et al., 2002; Shimokawa, 2002). Another type of ROCK inhibitor, 4-aminopyridine derivatives, is also widely used. Y-27632 is one typical example. Y-27632 inhibits both ROCK-I and ROCK-II through competitively binding to the ATP-binding site. However, because Y-27632 also inhibits PKA, PKC, and citron kinase (Ishizaki et al., 2000), researchers have attempted to optimize this compound to develop more potent and selective ROCK inhibitors. Y-39983 inhibits ROCK approximately 30 times more effectively than Y-27632 (Uehata et al., 1997; Tokushige et al., 2007). Y-39983 has been shown to decrease the intraocular pressure in the animal model of glaucoma (Nakajima et al., 2005; Tokushige et al., 2007), and promotes axonal regeneration after optic nerve injury (Sagawa et al., 2007).

## Therapeutic potential of RhoA/ROCK inhibitors

The concept of treating neurological disorders with RhoA/ROCK inhibitors is considered as a rational therapeutic approach. A phase I/IIa clinical trial has been completed for BA-210, the Rho inhibitor (Fehlings et al., 2011). This clinical trial was designed to investigate the effect of a single dose of BA-210 in patients with acute SCI. Sixteen patients with either cervical (C4–T1) or 32 patients with thoracic (T2–T12) SCI were treated within 7 days after injury with a single dose of 0.3, 1, 3, 6, or 9 mg of BA-210. All patients had acute, complete (i.e., American Spinal Injury Association impairment (ASIA) scale (Steeves et al., 2007) grade A) SCI. Inclusion criteria allowed both male and female subjects aged from 16 to 70 years. BA-210 was applied through a fibrin-mediated delivery system onto the dura matter at the lesion site of SCI. This was a relatively non-invasive delivery system, and it had been used for experimental studies in rodents (Guest et al., 1997; Kassam et al., 2004) and spinal surgery (Nakamura et al., 2005). The following tests and assessments were performed: vital signs, clinical laboratory tests, computed tomography (CT) scans of the spine, head, and abdomen, magnetic resonance imaging (MRI) of the spine, and ASIA assessment in the pre-study period and in the 1-year follow-up period after treatment. All doses were safe and well tolerated, and no severe adverse effects attributable to BA-210 were reported. ASIA assessment was used to evaluate the neurological status of patients. The results of this clinical trial suggest that the treatment of BA-210 increases neurological recovery in patients with both cervical and thoracic SCI (Fehlings et al., 2011). Changes in ASIA motor scores from baseline were larger in cervical patients (18.6 ± 19.3) than in thoracic patients (1.8 ± 5.1). The largest mean change in motor score was observed in patients with cervical SCI treated with 3 mg of BA-210. Although there was no placebo control group, this clinical trial demonstrates some possibilities of improvement of functional recovery by BA-210 treatment. Thus, RhoA/ROCK remains a promising molecular target for the treatment of neurological diseases.

## Conclusion

The evidence obtained from animal models and clinical trials implicate that inhibition of the RhoA/ROCK pathway would be an effective therapeutic approach for CNS disorders. However, unresolved problems should be addressed to achieve the therapeutic applications of RhoA/ROCK inhibitors. For example, timing of administration and low drug selectivity needs to be discussed in more detail. In the case of SCI, pharmacological treatments in rodents are often administered within 3 days of the injury (Rosenzweig and McDonald, 2004). However, delayed administration sometimes can promote recovery from SCI. Although immediate intrathecal treatment of the NgR competitive antagonist NEP1-40 has shown to improve axonal regrowth after hemisection injury (Grandpre et al., 2002), delayed treatment for up to 7 days after SCI also demonstrated anatomical and functional recovery (Li and Strittmatter, 2003). As mentioned above, the ROCK inhibitor fasudil inhibits both ROCK-I and ROCK-II. Since ROCK-I is mainly distributed in non-neuronal tissues, fasudil may cause some unwanted side effects or induce beneficial effects through glial cells (Tonges et al., 2011). Therefore, developing more selective inhibitors against ROCK-II will help to provide better therapeutic applications. Further studies will consolidate the evidence linking RhoA/ROCK inhibitors to neurological diseases.

### Conflict of interest statement

The authors declare that the research was conducted in the absence of any commercial or financial relationships that could be construed as a potential conflict of interest.

## References

[B1] Adelson J. D. Barreto G. E. Xu L. Kim T. Brott B. K. Ouyang Y. B.. (2012). Neuroprotection from stroke in the absence of MHCI or PirB. Neuron 73, 1100–1107. 10.1016/j.neuron.2012.01.020 22445338 PMC3314229

[B2] Anthony J. C. Breitner J. C. Zandi P. P. Meyer M. R. Jurasova I. Norton M. C.. (2000). Reduced prevalence of AD in users of NSAIDs and H2 receptor antagonists: the Cache County study. Neurology 54, 2066–2071. 10.1212/WNL.54.11.2066 10851364

[B3] Atwal J. K. Pinkston-Gosse J. Syken J. Stawicki S. Wu Y. Shatz C.. (2008). PirB is a functional receptor for myelin inhibitors of axonal regeneration. Science 322, 967–970. 10.1126/science.1161151 18988857

[B4] Barten L. J. Allington D. R. Procacci K. A. Rivey M. P. (2010). New approaches in the management of multiple sclerosis. Drug Des. Devel. Ther. 4, 343–366. 10.2147/DDDT.S9331 21151622 PMC2998807

[B5] Barton W. A. Liu B. P. Tzvetkova D. Jeffrey P. D. Fournier A. E. Sah D.. (2003). Structure and axon outgrowth inhibitor binding of the Nogo-66 receptor and related proteins. EMBO J. 22, 3291–3302. 10.1093/emboj/cdg325 12839991 PMC165649

[B6] Bartsch U. Bandtlow C. E. Schnell L. Bartsch S. Spillmann A. A. Rubin B. P.. (1995). Lack of evidence that myelin-associated glycoprotein is a major inhibitor of axonal regeneration in the CNS. Neuron 15, 1375–1381. 10.1016/0896-6273(95)90015-2 8845160

[B7] Beher D. Clarke E. E. Wrigley J. D. Martin A. C. Nadin A. Churcher I.. (2004). Selected non-steroidal anti-inflammatory drugs and their derivatives target gamma-secretase at a novel site. Evidence for an allosteric mechanism. J. Biol. Chem. 279, 43419–43426. 10.1074/jbc.M404937200 15304503

[B8] Benson M. D. Romero M. I. Lush M. E. Lu Q. R. Henkemeyer M. Parada L. F. (2005). Ephrin-B3 is a myelin-based inhibitor of neurite outgrowth. Proc. Natl. Acad. Sci. U.S.A. 102, 10694–10699. 10.1073/pnas.0504021102 16020529 PMC1175581

[B9] Bertrand J. Winton M. J. Rodriguez-Hernandez N. Campenot R. B. McKerracher L. (2005). Application of Rho antagonist to neuronal cell bodies promotes neurite growth in compartmented cultures and regeneration of retinal ganglion cell axons in the optic nerve of adult rats. J. Neurosci. 25, 1113–1121. 10.1523/JNEUROSCI.3931-04.2005 15689547 PMC6725958

[B10] Boato F. Hendrix S. Huelsenbeck S. C. Hofmann F. Grosse G. Djalali S.. (2010). C3 peptide enhances recovery from spinal cord injury by improved regenerative growth of descending fiber tracts. J. Cell Sci. 123, 1652–1662. 10.1242/jcs.066050 20406886

[B11] Borisoff J. F. Chan C. C. Hiebert G. W. Oschipok L. Robertson G. S. Zamboni R.. (2003). Suppression of Rho-kinase activity promotes axonal growth on inhibitory CNS substrates. Mol. Cell. Neurosci. 22, 405–416. 10.1016/S1044-7431(02)00032-5 12691741

[B12] Bowerman M. Beauvais A. Anderson C. L. Kothary R. (2010). Rho-kinase inactivation prolongs survival of an intermediate SMA mouse model. Hum. Mol. Genet. 19, 1468–1478. 10.1093/hmg/ddq021 20097679

[B13] Brabeck C. Beschorner R. Conrad S. Mittelbronn M. Bekure K. Meyermann R.. (2004). Lesional expression of RhoA and RhoB following traumatic brain injury in humans. J. Neurotrauma 21, 697–706. 10.1089/0897715041269597 15253798

[B14] Bregman B. S. Kunkel-Bagden E. Schnell L. Dai H. N. Gao D. Schwab M. E. (1995). Recovery from spinal cord injury mediated by antibodies to neurite growth inhibitors. Nature 378, 498–501. 10.1038/378498a0 7477407

[B15] Brosamle C. Huber A. B. Fiedler M. Skerra A. Schwab M. E. (2000). Regeneration of lesioned corticospinal tract fibers in the adult rat induced by a recombinant, humanized IN-1 antibody fragment. J. Neurosci. 20, 8061–8068. 11050127 10.1523/JNEUROSCI.20-21-08061.2000 PMC6772740

[B16] Brown B. A. Kantesaria P. P. McDevitt L. M. (2007). Fingolimod: a novel immunosuppressant for multiple sclerosis. Ann. Pharmacother. 41, 1660–1668. 10.1345/aph.1G424 17785617

[B17] Cafferty W. B. Duffy P. Huebner E. Strittmatter S. M. (2010). MAG and OMgp synergize with Nogo-A to restrict axonal growth and neurological recovery after spinal cord trauma. J. Neurosci. 30, 6825–6837. 10.1523/JNEUROSCI.6239-09.2010 20484625 PMC2883258

[B18] Cafferty W. B. Strittmatter S. M. (2006). The Nogo-Nogo receptor pathway limits a spectrum of adult CNS axonal growth. J. Neurosci. 26, 12242–12250. 10.1523/JNEUROSCI.3827-06.2006 17122049 PMC2848954

[B19] Cai D. Qiu J. Cao Z. McAtee M. Bregman B. S. Filbin M. T. (2001). Neuronal cyclic AMP controls the developmental loss in ability of axons to regenerate. J. Neurosci. 21, 4731–4739. 11425900 10.1523/JNEUROSCI.21-13-04731.2001 PMC6762375

[B20] Caroni P. Schwab M. E. (1988). Antibody against myelin-associated inhibitor of neurite growth neutralizes nonpermissive substrate properties of CNS white matter. Neuron 1, 85–96. 10.1016/0896-6273(88)90212-7 3272156

[B21] Chastain E. M. Duncan D. S. Rodgers J. M. Miller S. D. (2011). The role of antigen presenting cells in multiple sclerosis. Biochim. Biophys. Acta 1812, 265–274. 10.1016/j.bbadis.2010.07.008 20637861 PMC2970677

[B22] Chen H. Lin Y. Han M. Bai S. Wen S. (2010). Simultaneous quantitative analysis of fasudil and its active metabolite in human plasma by liquid chromatography electro-spray tandem mass spectrometry. J. Pharm. Biomed. Anal. 52, 242–248. 10.1016/j.jpba.2009.12.028 20080374

[B23] Chen M. S. Huber A. B. Van Der Haar M. E. Frank M. Schnell L. Spillmann A. A.. (2000). Nogo-A is a myelin-associated neurite outgrowth inhibitor and an antigen for monoclonal antibody IN-1. Nature 403, 434–439. 10.1038/35000219 10667796

[B24] Chivatakarn O. Kaneko S. He Z. Tessier-Lavigne M. Giger R. J. (2007). The Nogo-66 receptor NgR1 is required only for the acute growth cone-collapsing but not the chronic growth-inhibitory actions of myelin inhibitors. J. Neurosci. 27, 7117–7124. 10.1523/JNEUROSCI.1541-07.2007 17611264 PMC6794578

[B25] Davies S. P. Reddy H. Caivano M. Cohen P. (2000). Specificity and mechanism of action of some commonly used protein kinase inhibitors. Biochem. J. 351, 95–105. 10.1042/0264-6021:3510095 10998351 PMC1221339

[B26] Debellard M. E. Tang S. Mukhopadhyay G. Shen Y. J. Filbin M. T. (1996). Myelin-associated glycoprotein inhibits axonal regeneration from a variety of neurons via interaction with a sialoglycoprotein. Mol. Cell. Neurosci. 7, 89–101. 10.1006/mcne.1996.0007 8731478

[B27] Dergham P. Ellezam B. Essagian C. Avedissian H. Lubell W. D. McKerracher L. (2002). Rho signaling pathway targeted to promote spinal cord repair. J. Neurosci. 22, 6570–6577. 12151536 10.1523/JNEUROSCI.22-15-06570.2002 PMC6758168

[B28] Deyts C. Galan-Rodriguez B. Martin E. Bouveyron N. Roze E. Charvin D.. (2009). Dopamine D2 receptor stimulation potentiates PolyQ-Huntingtin-induced mouse striatal neuron dysfunctions via Rho/ROCK-II activation. PLoS ONE 4:e8287. 10.1371/journal.pone.0008287 20016831 PMC2790370

[B29] Dickendesher T. L. Baldwin K. T. Mironova Y. A. Koriyama Y. Raiker S. J. Askew K. L.. (2012). NgR1 and NgR3 are receptors for chondroitin sulfate proteoglycans. Nat. Neurosci. 15, 703–712. 10.1038/nn.3070 22406547 PMC3337880

[B30] Dimou L. Schnell L. Montani L. Duncan C. Simonen M. Schneider R.. (2006). Nogo-A-deficient mice reveal strain-dependent differences in axonal regeneration. J. Neurosci. 26, 5591–5603. 10.1523/JNEUROSCI.1103-06.2006 16723516 PMC6675256

[B31] Domeniconi M. Cao Z. Spencer T. Sivasankaran R. Wang K. Nikulina E.. (2002). Myelin-associated glycoprotein interacts with the Nogo66 receptor to inhibit neurite outgrowth. Neuron 35, 283–290. 10.1016/S0896-6273(02)00770-5 12160746

[B32] Dubreuil C. I. Winton M. J. McKerracher L. (2003). Rho activation patterns after spinal cord injury and the role of activated Rho in apoptosis in the central nervous system. J. Cell Biol. 162, 233–243. 10.1083/jcb.200301080 12860969 PMC2172802

[B33] Endo T. Spenger C. Tominaga T. Brene S. Olson L. (2007). Cortical sensory map rearrangement after spinal cord injury: fMRI responses linked to Nogo signalling. Brain 130, 2951–2961. 10.1093/brain/awm237 17913768

[B34] Eriksen J. L. Sagi S. A. Smith T. E. Weggen S. Das P. McLendon D. C.. (2003). NSAIDs and enantiomers of flurbiprofen target gamma-secretase and lower Abeta 42 *in vivo*. J. Clin. Invest. 112, 440–449. 10.1172/JCI18162 12897211 PMC166298

[B35] Esch F. S. Keim P. S. Beattie E. C. Blacher R. W. Culwell A. R. Oltersdorf T.. (1990). Cleavage of amyloid beta peptide during constitutive processing of its precursor. Science 248, 1122–1124. 10.1126/science.2111583 2111583

[B36] Fehlings M. G. Theodore N. Harrop J. Maurais G. Kuntz C. Shaffrey C. I.. (2011). A phase I/IIa clinical trial of a recombinant Rho protein antagonist in acute spinal cord injury. J. Neurotrauma 28, 787–796. 10.1089/neu.2011.1765 21381984

[B37] Filbin M. T. (2003). Myelin-associated inhibitors of axonal regeneration in the adult mammalian CNS. Nat. Rev. Neurosci. 4, 703–713. 10.1038/nrn1195 12951563

[B38] Fischer D. Petkova V. Thanos S. Benowitz L. I. (2004). Switching mature retinal ganglion cells to a robust growth state *in vivo*: gene expression and synergy with RhoA inactivation. J. Neurosci. 24, 8726–8740. 10.1523/JNEUROSCI.2774-04.2004 15470139 PMC6729954

[B39] Fisher D. Xing B. Dill J. Li H. Hoang H. H. Zhao Z.. (2011). Leukocyte common antigen-related phosphatase is a functional receptor for chondroitin sulfate proteoglycan axon growth inhibitors. J. Neurosci. 31, 14051–14066. 10.1523/JNEUROSCI.1737-11.2011 21976490 PMC3220601

[B40] Fournier A. E. Grandpre T. Strittmatter S. M. (2001). Identification of a receptor mediating Nogo-66 inhibition of axonal regeneration. Nature 409, 341–346. 10.1038/35053072 11201742

[B41] Fournier A. E. Takizawa B. T. Strittmatter S. M. (2003). Rho kinase inhibition enhances axonal regeneration in the injured CNS. J. Neurosci. 23, 1416–1423. 12598630 10.1523/JNEUROSCI.23-04-01416.2003 PMC6742251

[B42] Fujita Y. Endo S. Takai T. Yamashita T. (2011). Myelin suppresses axon regeneration by PIR-B/SHP-mediated inhibition of Trk activity. EMBO J. 30, 1389–1401. 10.1038/emboj.2011.55 21364532 PMC3094118

[B43] Fukata Y. Itoh T. J. Kimura T. Menager C. Nishimura T. Shiromizu T.. (2002). CRMP-2 binds to tubulin heterodimers to promote microtubule assembly. Nat. Cell Biol. 4, 583–591. 10.1038/ncb825 12134159

[B44] George R. Griffin J. W. (1994). Delayed macrophage responses and myelin clearance during Wallerian degeneration in the central nervous system: the dorsal radiculotomy model. Exp. Neurol. 129, 225–236. 10.1006/exnr.1994.1164 7957737

[B45] Gil V. Bichler Z. Lee J. K. Seira O. Llorens F. Bribian A.. (2010). Developmental expression of the oligodendrocyte myelin glycoprotein in the mouse telencephalon. Cereb. Cortex 20, 1769–1779. 10.1093/cercor/bhp246 19892785 PMC2901018

[B46] Grandpre T. Li S. Strittmatter S. M. (2002). Nogo-66 receptor antagonist peptide promotes axonal regeneration. Nature 417, 547–551. 10.1038/417547a 12037567

[B47] Grandpre T. Nakamura F. Vartanian T. Strittmatter S. M. (2000). Identification of the Nogo inhibitor of axon regeneration as a Reticulon protein. Nature 403, 439–444. 10.1038/35000226 10667797

[B48] Greenwood J. Walters C. E. Pryce G. Kanuga N. Beraud E. Baker D.. (2003). Lovastatin inhibits brain endothelial cell Rho-mediated lymphocyte migration and attenuates experimental autoimmune encephalomyelitis. FASEB J. 17, 905–907. 10.1096/fj.02-1014fje 12626426 PMC3831156

[B49] Griffin J. W. George R. Lobato C. Tyor W. R. Yan L. C. Glass J. D. (1992). Macrophage responses and myelin clearance during Wallerian degeneration: relevance to immune-mediated demyelination. J. Neuroimmunol. 40, 153–165. 10.1016/0165-5728(92)90129-9 1430148

[B50] Guermonprez P. Valladeau J. Zitvogel L. Thery C. Amigorena S. (2002). Antigen presentation and T cell stimulation by dendritic cells. Annu. Rev. Immunol. 20, 621–667. 10.1146/annurev.immunol.20.100301.064828 11861614

[B51] Guest J. D. Hesse D. Schnell L. Schwab M. E. Bunge M. B. Bunge R. P. (1997). Influence of IN-1 antibody and acidic FGF-fibrin glue on the response of injured corticospinal tract axons to human Schwann cell grafts. J. Neurosci. Res. 50, 888–905. 9418975 10.1002/(SICI)1097-4547(19971201)50:5<888::AID-JNR24>3.0.CO;2-W

[B52] Guo Q. Li S. Su B. (2007). Expression of oligodendrocyte myelin glycoprotein and its receptor NgR after the injury of rat central nervous system. Neurosci. Lett. 422, 103–108. 10.1016/j.neulet.2007.05.034 17630211

[B53] Habib A. A. Marton L. S. Allwardt B. Gulcher J. R. Mikol D. D. Hognason T.. (1998). Expression of the oligodendrocyte-myelin glycoprotein by neurons in the mouse central nervous system. J. Neurochem. 70, 1704–1711. 10.1046/j.1471-4159.1998.70041704.x 9523589

[B54] Hara M. Takayasu M. Watanabe K. Noda A. Takagi T. Suzuki Y.. (2000). Protein kinase inhibition by fasudil hydrochloride promotes neurological recovery after spinal cord injury in rats. J. Neurosurg. 93, 94–101. 10.3171/spi.2000.93.1.0094 10879764

[B55] Harrington A. W. Li Q. M. Tep C. Park J. B. He Z. Yoon S. O. (2008). The role of Kalirin9 in p75/nogo receptor-mediated RhoA activation in cerebellar granule neurons. J. Biol. Chem. 283, 24690–24697. 10.1074/jbc.M802188200 18625710 PMC2529002

[B56] Hata K. Fujitani M. Yasuda Y. Doya H. Saito T. Yamagishi S.. (2006). RGMa inhibition promotes axonal growth and recovery after spinal cord injury. J. Cell Biol. 173, 47–58. 10.1083/jcb.200508143 16585268 PMC2063787

[B57] Hata K. Kaibuchi K. Inagaki S. Yamashita T. (2009). Unc5B associates with LARG to mediate the action of repulsive guidance molecule. J. Cell Biol. 184, 737–750. 10.1083/jcb.200807029 19273616 PMC2686409

[B58] Hauser S. L. Oksenberg J. R. (2006). The neurobiology of multiple sclerosis: genes, inflammation, and neurodegeneration. Neuron 52, 61–76. 10.1016/j.neuron.2006.09.011 17015227

[B59] He Z. Koprivica V. (2004). The Nogo signaling pathway for regeneration block. Annu. Rev. Neurosci. 27, 341–368. 10.1146/annurev.neuro.27.070203.144340 15217336

[B60] Hendriks J. J. Alblas J. Van Der Pol S. M. Van Tol E. A. Dijkstra C. D. De Vries H. E. (2004). Flavonoids influence monocytic GTPase activity and are protective in experimental allergic encephalitis. J. Exp. Med. 200, 1667–1672. 10.1084/jem.20040819 15611292 PMC2212002

[B61] Hilas O. Patel P. N. Lam S. (2010). Disease modifying agents for multiple sclerosis. Open Neurol. J. 4, 15–24. 10.2174/1874205X01004010015 21258574 PMC3024587

[B62] Hou S. W. Liu C. Y. Li Y. H. Yu J. Z. Feng L. Liu Y. T.. (2012). Fasudil ameliorates disease progression in experimental autoimmune encephalomyelitis, acting possibly through antiinflammatory effect. CNS Neurosci. Ther. 18, 909–917. 10.1111/cns.12002 22994384 PMC6493591

[B63] Hsieh S. H. Ferraro G. B. Fournier A. E. (2006). Myelin-associated inhibitors regulate cofilin phosphorylation and neuronal inhibition through LIM kinase and Slingshot phosphatase. J. Neurosci. 26, 1006–1015. 10.1523/JNEUROSCI.2806-05.2006 16421320 PMC6675360

[B64] Hu J. H. Chernoff K. Pelech S. Krieger C. (2003). Protein kinase and protein phosphatase expression in the central nervous system of G93A mSOD over-expressing mice. J. Neurochem. 85, 422–431. 10.1046/j.1471-4159.2003.01669.x 12675918

[B65] Huber A. B. Weinmann O. Brosamle C. Oertle T. Schwab M. E. (2002). Patterns of Nogo mRNA and protein expression in the developing and adult rat and after CNS lesions. J. Neurosci. 22, 3553–3567. 11978832 10.1523/JNEUROSCI.22-09-03553.2002 PMC6758364

[B66] Inagaki N. Chihara K. Arimura N. Menager C. Kawano Y. Matsuo N.. (2001). CRMP-2 induces axons in cultured hippocampal neurons. Nat. Neurosci. 4, 781–782. 10.1038/90476 11477421

[B67] Ishida T. Takanashi Y. Kiwada H. (2006). Safe and efficient drug delivery system with liposomes for intrathecal application of an antivasospastic drug, fasudil. Biol. Pharm. Bull. 29, 397–402. 10.1248/bpb.29.397 16508135

[B68] Ishizaki T. Uehata M. Tamechika I. Keel J. Nonomura K. Maekawa M.. (2000). Pharmacological properties of Y-27632, a specific inhibitor of rho-associated kinases. Mol. Pharmacol. 57, 976–983. 10779382

[B69] Jaffe A. B. Hall A. (2005). Rho GTPases: biochemistry and biology. Annu. Rev. Cell Dev. Biol. 21, 247–269. 10.1146/annurev.cellbio.21.020604.150721 16212495

[B70] Ji B. Case L. C. Liu K. Shao Z. Lee X. Yang Z.. (2008). Assessment of functional recovery and axonal sprouting in oligodendrocyte-myelin glycoprotein (OMgp) null mice after spinal cord injury. Mol. Cell. Neurosci. 39, 258–267. 10.1016/j.mcn.2008.07.004 18692574 PMC2646371

[B71] Johnson P. W. Abramow-Newerly W. Seilheimer B. Sadoul R. Tropak M. B. Arquint M.. (1989). Recombinant myelin-associated glycoprotein confers neural adhesion and neurite outgrowth function. Neuron 3, 377–385. 10.1016/0896-6273(89)90262-6 2484339

[B72] Jones L. L. Margolis R. U. Tuszynski M. H. (2003). The chondroitin sulfate proteoglycans neurocan, brevican, phosphacan, and versican are differentially regulated following spinal cord injury. Exp. Neurol. 182, 399–411. 10.1016/S0014-4886(03)00087-6 12895450

[B73] Jones M. V. Nguyen T. T. Ewaleifoh O. Lebson L. Whartenby K. A. Griffin J. W.. (2013). Accelerated axon loss in MOG35-55 experimental autoimmune encephalomyelitis (EAE) in myelin-associated glycoprotein-deficient (MAGKO) mice. J. Neuroimmunol. 262, 53–61. 10.1016/j.jneuroim.2013.06.008 23899666

[B74] Kassam A. Nemoto E. Balzer J. Rao G. Welch W. C. Kuwabara H.. (2004). Effects of Tisseel fibrin glue on the central nervous system of nonhuman primates. Ear. Nose. Throat J. 83, 246–248, 250, 252 passim. 15147095

[B75] Kempf A. Tews B. Arzt M. E. Weinmann O. Obermair F. J. Pernet V.. (2014). The sphingolipid receptor S1PR2 is a receptor for Nogo-a repressing synaptic plasticity. PLoS Biol. 12:e1001763. 10.1371/journal.pbio.1001763 24453941 PMC3891622

[B76] Kim J. E. Li S. Grandpre T. Qiu D. Strittmatter S. M. (2003). Axon regeneration in young adult mice lacking Nogo-A/B. Neuron 38, 187–199. 10.1016/S0896-6273(03)00147-8 12718854

[B77] Kim J. E. Liu B. P. Park J. H. Strittmatter S. M. (2004). Nogo-66 receptor prevents raphespinal and rubrospinal axon regeneration and limits functional recovery from spinal cord injury. Neuron 44, 439–451. 10.1016/j.neuron.2004.10.015 15504325

[B78] Kinter J. Lazzati T. Schmid D. Zeis T. Erne B. Lutzelschwab R.. (2012). An essential role of MAG in mediating axon-myelin attachment in Charcot-Marie-Tooth 1A disease. Neurobiol. Dis. 49C, 221–231. 10.1016/j.nbd.2012.08.009 22940629 PMC3612363

[B79] Kottis V. Thibault P. Mikol D. Xiao Z. C. Zhang R. Dergham P.. (2002). Oligodendrocyte-myelin glycoprotein (OMgp) is an inhibitor of neurite outgrowth. J. Neurochem. 82, 1566–1569. 10.1046/j.1471-4159.2002.01146.x 12354307

[B80] Krumbholz M. Meinl E. (2014). B cells in MS and NMO: pathogenesis and therapy. Semin. Immunopathol. 36, 339–350. 10.1007/s00281-014-0424-x 24832354

[B81] Kubo T. Endo M. Hata K. Taniguchi J. Kitajo K. Tomura S.. (2008). Myosin IIA is required for neurite outgrowth inhibition produced by repulsive guidance molecule. J. Neurochem. 105, 113–126. 10.1111/j.1471-4159.2007.05125.x 18005226

[B82] Kurihara Y. Arie Y. Iketani M. Ito H. Nishiyama K. Sato Y.. (2012). The carboxyl-terminal region of Crtac1B/LOTUS acts as a functional domain in endogenous antagonism to Nogo receptor-1. Biochem. Biophys. Res. Commun. 418, 390–395. 10.1016/j.bbrc.2012.01.033 22281491

[B83] Laufs U. Endres M. Stagliano N. Amin-Hanjani S. Chui D. S. Yang S. X.. (2000). Neuroprotection mediated by changes in the endothelial actin cytoskeleton. J. Clin. Invest. 106, 15–24. 10.1172/JCI9639 10880044 PMC314365

[B84] Lauren J. Airaksinen M. S. Saarma M. Timmusk T. (2003). Two novel mammalian Nogo receptor homologs differentially expressed in the central and peripheral nervous systems. Mol. Cell. Neurosci. 24, 581–594. 10.1016/S1044-7431(03)00199-4 14664809

[B85] Lee H. Raiker S. J. Venkatesh K. Geary R. Robak L. A. Zhang Y.. (2008). Synaptic function for the Nogo-66 receptor NgR1: regulation of dendritic spine morphology and activity-dependent synaptic strength. J. Neurosci. 28, 2753–2765. 10.1523/JNEUROSCI.5586-07.2008 18337405 PMC6670664

[B86] Lee J. K. Geoffroy C. G. Chan A. F. Tolentino K. E. Crawford M. J. Leal M. A.. (2010). Assessing spinal axon regeneration and sprouting in Nogo-, MAG-, and OMgp-deficient mice. Neuron 66, 663–670. 10.1016/j.neuron.2010.05.002 20547125 PMC2896331

[B87] Lee J. K. Kim J. E. Sivula M. Strittmatter S. M. (2004). Nogo receptor antagonism promotes stroke recovery by enhancing axonal plasticity. J. Neurosci. 24, 6209–6217. 10.1523/JNEUROSCI.1643-04.2004 15240813 PMC6729662

[B88] Lee X. Hu Y. Zhang Y. Yang Z. Shao Z. Qiu M.. (2011). Oligodendrocyte differentiation and myelination defects in OMgp null mice. Mol. Cell. Neurosci. 46, 752–761. 10.1016/j.mcn.2011.02.008 21352918

[B89] Lehmann M. Fournier A. Selles-Navarro I. Dergham P. Sebok A. Leclerc N.. (1999). Inactivation of Rho signaling pathway promotes CNS axon regeneration. J. Neurosci. 19, 7537–7547. 10460260 10.1523/JNEUROSCI.19-17-07537.1999 PMC6782492

[B90] Li S. Strittmatter S. M. (2003). Delayed systemic Nogo-66 receptor antagonist promotes recovery from spinal cord injury. J. Neurosci. 23, 4219–4227. 12764110 10.1523/JNEUROSCI.23-10-04219.2003 PMC6741116

[B91] Lingor P. Teusch N. Schwarz K. Mueller R. Mack H. Bahr M.. (2007). Inhibition of Rho kinase (ROCK) increases neurite outgrowth on chondroitin sulphate proteoglycan *in vitro* and axonal regeneration in the adult optic nerve *in vivo*. J. Neurochem. 103, 181–189. 10.1111/j.1471-4159.2007.04756.x 17608642

[B92] Lingor P. Tonges L. Pieper N. Bermel C. Barski E. Planchamp V.. (2008). ROCK inhibition and CNTF interact on intrinsic signalling pathways and differentially regulate survival and regeneration in retinal ganglion cells. Brain 131, 250–263. 10.1093/brain/awm284 18063589

[B93] Liu B. P. Fournier A. Grandpre T. Strittmatter S. M. (2002). Myelin-associated glycoprotein as a functional ligand for the Nogo-66 receptor. Science 297, 1190–1193. 10.1126/science.1073031 12089450

[B94] Lord-Fontaine S. Yang F. Diep Q. Dergham P. Munzer S. Tremblay P.. (2008). Local inhibition of Rho signaling by cell-permeable recombinant protein BA-210 prevents secondary damage and promotes functional recovery following acute spinal cord injury. J. Neurotrauma 25, 1309–1322. 10.1089/neu.2008.0613 19061375

[B95] Madura T. Yamashita T. Kubo T. Fujitani M. Hosokawa K. Tohyama M. (2004). Activation of Rho in the injured axons following spinal cord injury. EMBO Rep. 5, 412–417. 10.1038/sj.embor.7400117 15031718 PMC1299028

[B96] McGee A. W. Yang Y. Fischer Q. S. Daw N. W. Strittmatter S. M. (2005). Experience-driven plasticity of visual cortex limited by myelin and Nogo receptor. Science 309, 2222–2226. 10.1126/science.1114362 16195464 PMC2856689

[B97] McGeer P. L. Schulzer M. McGeer E. G. (1996). Arthritis and anti-inflammatory agents as possible protective factors for Alzheimer's disease: a review of 17 epidemiologic studies. Neurology 47, 425–432. 10.1212/WNL.47.2.425 8757015

[B98] McKeon R. J. Jurynec M. J. Buck C. R. (1999). The chondroitin sulfate proteoglycans neurocan and phosphacan are expressed by reactive astrocytes in the chronic CNS glial scar. J. Neurosci. 19, 10778–10788. 10594061 10.1523/JNEUROSCI.19-24-10778.1999 PMC6784959

[B99] McKerracher L. David S. Jackson D. L. Kottis V. Dunn R. J. Braun P. E. (1994). Identification of myelin-associated glycoprotein as a major myelin-derived inhibitor of neurite growth. Neuron 13, 805–811. 10.1016/0896-6273(94)90247-X 7524558

[B100] Merkler D. Metz G. A. Raineteau O. Dietz V. Schwab M. E. Fouad K. (2001). Locomotor recovery in spinal cord-injured rats treated with an antibody neutralizing the myelin-associated neurite growth inhibitor Nogo-A. J. Neurosci. 21, 3665–3673. 11331396 10.1523/JNEUROSCI.21-10-03665.2001 PMC6762500

[B101] Mi S. Lee X. Shao Z. Thill G. Ji B. Relton J.. (2004). LINGO-1 is a component of the Nogo-66 receptor/p75 signaling complex. Nat. Neurosci. 7, 221–228. 10.1038/nn1188 14966521

[B102] Mimura F. Yamagishi S. Arimura N. Fujitani M. Kubo T. Kaibuchi K.. (2006). Myelin-associated glycoprotein inhibits microtubule assembly by a Rho-kinase-dependent mechanism. J. Biol. Chem. 281, 15970–15979. 10.1074/jbc.M510934200 16595691

[B103] Monnier P. P. Sierra A. Schwab J. M. Henke-Fahle S. Mueller B. K. (2003). The Rho/ROCK pathway mediates neurite growth-inhibitory activity associated with the chondroitin sulfate proteoglycans of the CNS glial scar. Mol. Cell. Neurosci. 22, 319–330. 10.1016/S1044-7431(02)00035-0 12691734

[B104] Moreau-Fauvarque C. Kumanogoh A. Camand E. Jaillard C. Barbin G. Boquet I.. (2003). The transmembrane semaphorin Sema4D/CD100, an inhibitor of axonal growth, is expressed on oligodendrocytes and upregulated after CNS lesion. J. Neurosci. 23, 9229–9239. 14534257 10.1523/JNEUROSCI.23-27-09229.2003 PMC6740837

[B105] Mueller B. K. Mack H. Teusch N. (2005). Rho kinase, a promising drug target for neurological disorders. Nat. Rev. Drug Discov. 4, 387–398. 10.1038/nrd1719 15864268

[B106] Mueller B. K. Yamashita T. Schaffar G. Mueller R. (2006). The role of repulsive guidance molecules in the embryonic and adult vertebrate central nervous system. Philos. Trans. R. Soc. Lond. B Biol. Sci. 361, 1513–1529. 10.1098/rstb.2006.1888 16939972 PMC1664662

[B107] Mukhopadhyay G. Doherty P. Walsh F. S. Crocker P. R. Filbin M. T. (1994). A novel role for myelin-associated glycoprotein as an inhibitor of axonal regeneration. Neuron 13, 757–767. 10.1016/0896-6273(94)90042-6 7522484

[B108] Muramatsu R. Kubo T. Mori M. Nakamura Y. Fujita Y. Akutsu T.. (2011). RGMa modulates T cell responses and is involved in autoimmune encephalomyelitis. Nat. Med. 17, 488–494. 10.1038/nm.2321 21423182

[B109] Nakagawa O. Fujisawa K. Ishizaki T. Saito Y. Nakao K. Narumiya S. (1996). ROCK-I and ROCK-II, two isoforms of Rho-associated coiled-coil forming protein serine/threonine kinase in mice. FEBS Lett. 392, 189–193. 10.1016/0014-5793(96)00811-3 8772201

[B110] Nakajima E. Nakajima T. Minagawa Y. Shearer T. R. Azuma M. (2005). Contribution of ROCK in contraction of trabecular meshwork: proposed mechanism for regulating aqueous outflow in monkey and human eyes. J. Pharm. Sci. 94, 701–708. 10.1002/jps.20285 15682386

[B111] Nakamura H. Matsuyama Y. Yoshihara H. Sakai Y. Katayama Y. Nakashima S.. (2005). The effect of autologous fibrin tissue adhesive on postoperative cerebrospinal fluid leak in spinal cord surgery: a randomized controlled trial. Spine (Phila Pa 1976) 30, E347–E351. 10.1097/01.brs.0000167820.54413.8e 15990651

[B112] Nakamura Y. Fujita Y. Ueno M. Takai T. Yamashita T. (2011). Paired immunoglobulin-like receptor B knockout does not enhance axonal regeneration or locomotor recovery after spinal cord injury. J. Biol. Chem. 286, 1876–1883. 10.1074/jbc.M110.163493 21087927 PMC3023483

[B113] Neuhaus O. Stuve O. Zamvil S. S. Hartung H. P. (2004). Are statins a treatment option for multiple sclerosis? Lancet Neurol. 3, 369–371. 10.1016/S1474-4422(04)00770-7 15157852

[B114] Nguyen T. Mehta N. R. Conant K. Kim K. J. Jones M. Calabresi P. A.. (2009). Axonal protective effects of the myelin-associated glycoprotein. J. Neurosci. 29, 630–637. 10.1523/JNEUROSCI.5204-08.2009 19158290 PMC2774126

[B115] Noseworthy J. H. Lucchinetti C. Rodriguez M. Weinshenker B. G. (2000). Multiple sclerosis. N. Engl. J. Med. 343, 938–952. 10.1056/NEJM200009283431307 11006371

[B116] Novotny-Diermayr V. Lin B. Gu L. Cao X. (2005). Modulation of the interleukin-6 receptor subunit glycoprotein 130 complex and its signaling by LMO4 interaction. J. Biol. Chem. 280, 12747–12757. 10.1074/jbc.M500175200 15677447

[B117] Ohashi K. Nagata K. Maekawa M. Ishizaki T. Narumiya S. Mizuno K. (2000). Rho-associated kinase ROCK activates LIM-kinase 1 by phosphorylation at threonine 508 within the activation loop. J. Biol. Chem. 275, 3577–3582. 10.1074/jbc.275.5.3577 10652353

[B118] Omoto S. Ueno M. Mochio S. Takai T. Yamashita T. (2010). Genetic deletion of paired immunoglobulin-like receptor B does not promote axonal plasticity or functional recovery after traumatic brain injury. J. Neurosci. 30, 13045–13052. 10.1523/JNEUROSCI.3228-10.2010 20881122 PMC6633514

[B119] Park J. H. Gimbel D. A. Grandpre T. Lee J. K. Kim J. E. Li W.. (2006). Alzheimer precursor protein interaction with the Nogo-66 receptor reduces amyloid-beta plaque deposition. J. Neurosci. 26, 1386–1395. 10.1523/JNEUROSCI.3291-05.2006 16452662 PMC2846286

[B120] Pedrini S. Carter T. L. Prendergast G. Petanceska S. Ehrlich M. E. Gandy S. (2005). Modulation of statin-activated shedding of Alzheimer APP ectodomain by ROCK. PLoS Med. 2:e18. 10.1371/journal.pmed.0020018 15647781 PMC543463

[B121] Pignot V. Hein A. E. Barske C. Wiessner C. Walmsley A. R. Kaupmann K.. (2003). Characterization of two novel proteins, NgRH1 and NgRH2, structurally and biochemically homologous to the Nogo-66 receptor. J. Neurochem. 85, 717–728. 10.1046/j.1471-4159.2003.01710.x 12694398

[B122] Prinjha R. Moore S. E. Vinson M. Blake S. Morrow R. Christie G.. (2000). Inhibitor of neurite outgrowth in humans. Nature 403, 383–384. 10.1038/35000287 10667780

[B123] Quarles R. H. (2007). Myelin-associated glycoprotein (MAG): past, present and beyond. J. Neurochem. 100, 1431–1448. 10.1111/j.1471-4159.2006.04319.x 17241126

[B124] Riento K. Ridley A. J. (2003). Rocks: multifunctional kinases in cell behaviour. Nat. Rev. Mol. Cell Biol. 4, 446–456. 10.1038/nrm1128 12778124

[B125] Rikitake Y. Kim H. H. Huang Z. Seto M. Yano K. Asano T.. (2005). Inhibition of Rho kinase (ROCK) leads to increased cerebral blood flow and stroke protection. Stroke 36, 2251–2257. 10.1161/01.STR.0000181077.84981.11 16141422 PMC2633591

[B126] Rosenzweig E. S. McDonald J. W. (2004). Rodent models for treatment of spinal cord injury: research trends and progress toward useful repair. Curr. Opin. Neurol. 17, 121–131. 10.1097/00019052-200404000-00007 15021237

[B127] Sagawa H. Terasaki H. Nakamura M. Ichikawa M. Yata T. Tokita Y.. (2007). A novel ROCK inhibitor, Y-39983, promotes regeneration of crushed axons of retinal ganglion cells into the optic nerve of adult cats. Exp. Neurol. 205, 230–240. 10.1016/j.expneurol.2007.02.002 17359977

[B128] Salzer J. L. Pedraza L. Brown M. Struyk A. Afar D. Bell J. (1990). Structure and function of the myelin-associated glycoproteins. Ann. N.Y. Acad. Sci. 605, 302–312. 10.1111/j.1749-6632.1990.tb42404.x 1702604

[B129] Sasaki Y. Suzuki M. Hidaka H. (2002). The novel and specific Rho-kinase inhibitor (S)-(+)-2-methyl-1-[(4-methyl-5-isoquinoline)sulfonyl]-homopiperazine as a probing molecule for Rho-kinase-involved pathway. Pharmacol. Ther. 93, 225–232. 10.1016/S0163-7258(02)00191-2 12191614

[B130] Sato Y. Iketani M. Kurihara Y. Yamaguchi M. Yamashita N. Nakamura F.. (2011). Cartilage acidic protein-1B (LOTUS), an endogenous Nogo receptor antagonist for axon tract formation. Science 333, 769–773. 10.1126/science.1204144 21817055 PMC3244695

[B131] Satoh S. Utsunomiya T. Tsurui K. Kobayashi T. Ikegaki I. Sasaki Y.. (2001). Pharmacological profile of hydroxy fasudil as a selective rho kinase inhibitor on ischemic brain damage. Life Sci. 69, 1441–1453. 10.1016/S0024-3205(01)01229-2 11531167

[B132] Schaffar G. Taniguchi J. Brodbeck T. Meyer A. H. Schmidt M. Yamashita T.. (2008). LIM-only protein 4 interacts directly with the repulsive guidance molecule A receptor Neogenin. J. Neurochem. 107, 418–431. 10.1111/j.1471-4159.2008.05621.x 18702663

[B133] Schnell L. Schwab M. E. (1990). Axonal regeneration in the rat spinal cord produced by an antibody against myelin-associated neurite growth inhibitors. Nature 343, 269–272. 10.1038/343269a0 2300171

[B134] Schwab J. M. Monnier P. P. Schluesener H. J. Conrad S. Beschorner R. Chen L.. (2005). Central nervous system injury-induced repulsive guidance molecule expression in the adult human brain. Arch. Neurol. 62, 1561–1568. 10.1001/archneur.62.10.1561 16216939

[B135] Schwab M. E. Bartholdi D. (1996). Degeneration and regeneration of axons in the lesioned spinal cord. Physiol. Rev. 76, 319–370. 8618960 10.1152/physrev.1996.76.2.319

[B136] Shao J. Welch W. J. Diprospero N. A. Diamond M. I. (2008). Phosphorylation of profilin by ROCK1 regulates polyglutamine aggregation. Mol. Cell. Biol. 28, 5196–5208. 10.1128/MCB.00079-08 18573880 PMC2519718

[B137] Shen Y. Tenney A. P. Busch S. A. Horn K. P. Cuascut F. X. Liu K.. (2009). PTPsigma is a receptor for chondroitin sulfate proteoglycan, an inhibitor of neural regeneration. Science 326, 592–596. 10.1126/science.1178310 19833921 PMC2811318

[B138] Shimokawa H. (2002). Rho-kinase as a novel therapeutic target in treatment of cardiovascular diseases. J. Cardiovasc. Pharmacol. 39, 319–327. 10.1097/00005344-200203000-00001 11862109

[B139] Simonen M. Pedersen V. Weinmann O. Schnell L. Buss A. Ledermann B.. (2003). Systemic deletion of the myelin-associated outgrowth inhibitor Nogo-A improves regenerative and plastic responses after spinal cord injury. Neuron 38, 201–211. 10.1016/S0896-6273(03)00226-5 12718855

[B140] Sisodia S. S. Koo E. H. Beyreuther K. Unterbeck A. Price D. L. (1990). Evidence that beta-amyloid protein in Alzheimer's disease is not derived by normal processing. Science 248, 492–495. 10.1126/science.1691865 1691865

[B141] Steeves J. D. Lammertse D. Curt A. Fawcett J. W. Tuszynski M. H. Ditunno J. F.. (2007). Guidelines for the conduct of clinical trials for spinal cord injury (SCI) as developed by the ICCP panel: clinical trial outcome measures. Spinal Cord 45, 206–221. 10.1038/sj.sc.3102008 17179972

[B142] Sumi T. Matsumoto K. Nakamura T. (2001). Specific activation of LIM kinase 2 via phosphorylation of threonine 505 by ROCK, a Rho-dependent protein kinase. J. Biol. Chem. 276, 670–676. 10.1074/jbc.M007074200 11018042

[B143] Sun X. Minohara M. Kikuchi H. Ishizu T. Tanaka M. Piao H.. (2006). The selective Rho-kinase inhibitor Fasudil is protective and therapeutic in experimental autoimmune encephalomyelitis. J. Neuroimmunol. 180, 126–134. 10.1016/j.jneuroim.2006.06.027 16996142

[B144] Sung J. K. Miao L. Calvert J. W. Huang L. Louis Harkey H. Zhang J. H. (2003). A possible role of RhoA/Rho-kinase in experimental spinal cord injury in rat. Brain Res. 959, 29–38. 10.1016/S0006-8993(02)03717-4 12480155

[B145] Sunico C. R. Dominguez G. Garcia-Verdugo J. M. Osta R. Montero F. Moreno-Lopez B. (2011). Reduction in the motoneuron inhibitory/excitatory synaptic ratio in an early-symptomatic mouse model of amyotrophic lateral sclerosis. Brain Pathol. 21, 1–15. 10.1111/j.1750-3639.2010.00417.x 20653686 PMC8094302

[B146] Syken J. Grandpre T. Kanold P. O. Shatz C. J. (2006). PirB restricts ocular-dominance plasticity in visual cortex. Science 313, 1795–1800. 10.1126/science.1128232 16917027

[B147] Takahashi Y. Hayashi I. Tominari Y. Rikimaru K. Morohashi Y. Kan T.. (2003). Sulindac sulfide is a noncompetitive gamma-secretase inhibitor that preferentially reduces Abeta 42 generation. J. Biol. Chem. 278, 18664–18670. 10.1074/jbc.M301619200 12637581

[B148] Takai T. (2005). Paired immunoglobulin-like receptors and their MHC class I recognition. Immunology 115, 433–440. 10.1111/j.1365-2567.2005.02177.x 16011512 PMC1782189

[B149] Tanaka H. Yamashita T. Yachi K. Fujiwara T. Yoshikawa H. Tohyama M. (2004). Cytoplasmic p21(Cip1/WAF1) enhances axonal regeneration and functional recovery after spinal cord injury in rats. Neuroscience 127, 155–164. 10.1016/j.neuroscience.2004.05.010 15219678

[B150] Tang X. Davies J. E. Davies S. J. (2003). Changes in distribution, cell associations, and protein expression levels of NG2, neurocan, phosphacan, brevican, versican V2, and tenascin-C during acute to chronic maturation of spinal cord scar tissue. J. Neurosci. Res. 71, 427–444. 10.1002/jnr.10523 12526031

[B151] Tokushige H. Inatani M. Nemoto S. Sakaki H. Katayama K. Uehata M.. (2007). Effects of topical administration of y-39983, a selective rho-associated protein kinase inhibitor, on ocular tissues in rabbits and monkeys. Invest. Ophthalmol. Vis. Sci. 48, 3216–3222. 10.1167/iovs.05-1617 17591891

[B152] Tompkins S. M. Padilla J. Dal Canto M. C. Ting J. P. Van Kaer L. Miller S. D. (2002). De novo central nervous system processing of myelin antigen is required for the initiation of experimental autoimmune encephalomyelitis. J. Immunol. 168, 4173–4183. 10.4049/jimmunol.168.8.4173 11937578

[B153] Tonges L. Frank T. Tatenhorst L. Saal K. A. Koch J. C. Szego E. M.. (2012). Inhibition of rho kinase enhances survival of dopaminergic neurons and attenuates axonal loss in a mouse model of Parkinson's disease. Brain 135, 3355–3370. 10.1093/brain/aws254 23087045 PMC3501973

[B154] Tonges L. Koch J. C. Bahr M. Lingor P. (2011). ROCKing regeneration: Rho Kinase inhibition as molecular target for neurorestoration. Front. Mol. Neurosci. 4:39. 10.3389/fnmol.2011.00039 22065949 PMC3207219

[B155] Toshima Y. Satoh S. Ikegaki I. Asano T. (2000). A new model of cerebral microthrombosis in rats and the neuroprotective effect of a Rho-kinase inhibitor. Stroke 31, 2245–2250. 10.1161/01.STR.31.9.2245 10978059

[B156] Trapp B. D. Nave K. A. (2008). Multiple sclerosis: an immune or neurodegenerative disorder? Annu. Rev. Neurosci. 31, 247–269. 10.1146/annurev.neuro.30.051606.094313 18558855

[B157] Turnley A. M. Bartlett P. F. (1998). MAG and MOG enhance neurite outgrowth of embryonic mouse spinal cord neurons. Neuroreport 9, 1987–1990. 10.1097/00001756-199806220-00013 9674579

[B158] Uehata M. Ishizaki T. Satoh H. Ono T. Kawahara T. Morishita T.. (1997). Calcium sensitization of smooth muscle mediated by a Rho-associated protein kinase in hypertension. Nature 389, 990–994. 10.1038/40187 9353125

[B159] Venkatesh K. Chivatakarn O. Lee H. Joshi P. S. Kantor D. B. Newman B. A.. (2005). The Nogo-66 receptor homolog NgR2 is a sialic acid-dependent receptor selective for myelin-associated glycoprotein. J. Neurosci. 25, 808–822. 10.1523/JNEUROSCI.4464-04.2005 15673660 PMC6725623

[B160] Walters C. E. Pryce G. Hankey D. J. Sebti S. M. Hamilton A. D. Baker D.. (2002). Inhibition of Rho GTPases with protein prenyltransferase inhibitors prevents leukocyte recruitment to the central nervous system and attenuates clinical signs of disease in an animal model of multiple sclerosis. J. Immunol. 168, 4087–4094. 10.4049/jimmunol.168.8.4087 11937568 PMC3836400

[B161] Wang K. C. Kim J. A. Sivasankaran R. Segal R. He Z. (2002a). P75 interacts with the Nogo receptor as a co-receptor for Nogo, MAG and OMgp. Nature 420, 74–78. 10.1038/nature01176 12422217

[B162] Wang K. C. Koprivica V. Kim J. A. Sivasankaran R. Guo Y. Neve R. L.. (2002b). Oligodendrocyte-myelin glycoprotein is a Nogo receptor ligand that inhibits neurite outgrowth. Nature 417, 941–944. 10.1038/nature00867 12068310

[B163] Wang X. Chun S. J. Treloar H. Vartanian T. Greer C. A. Strittmatter S. M. (2002c). Localization of Nogo-A and Nogo-66 receptor proteins at sites of axon-myelin and synaptic contact. J. Neurosci. 22, 5505–5515. 12097502 10.1523/JNEUROSCI.22-13-05505.2002 PMC6758202

[B164] Weggen S. Eriksen J. L. Das P. Sagi S. A. Wang R. Pietrzik C. U.. (2001). A subset of NSAIDs lower amyloidogenic Abeta42 independently of cyclooxygenase activity. Nature 414, 212–216. 10.1038/35102591 11700559

[B165] Wiessner C. Bareyre F. M. Allegrini P. R. Mir A. K. Frentzel S. Zurini M.. (2003). Anti-Nogo-A antibody infusion 24 hours after experimental stroke improved behavioral outcome and corticospinal plasticity in normotensive and spontaneously hypertensive rats. J. Cereb. Blood Flow Metab. 23, 154–165. 10.1097/00004647-200302000-00003 12571447

[B166] Wills Z. P. Mandel-Brehm C. Mardinly A. R. McCord A. E. Giger R. J. Greenberg M. E. (2012). The nogo receptor family restricts synapse number in the developing hippocampus. Neuron 73, 466–481. 10.1016/j.neuron.2011.11.029 22325200 PMC3532882

[B167] Wong S. T. Henley J. R. Kanning K. C. Huang K. H. Bothwell M. Poo M. M. (2002). A p75(NTR) and Nogo receptor complex mediates repulsive signaling by myelin-associated glycoprotein. Nat. Neurosci. 5, 1302–1308. 10.1038/nn975 12426574

[B168] Worter V. Schweigreiter R. Kinzel B. Mueller M. Barske C. Bock G.. (2009). Inhibitory activity of myelin-associated glycoprotein on sensory neurons is largely independent of NgR1 and NgR2 and resides within Ig-Like domains 4 and 5. PLoS ONE 4:e5218. 10.1371/journal.pone.0005218 19367338 PMC2666269

[B169] Yamashita T. Higuchi H. Tohyama M. (2002). The p75 receptor transduces the signal from myelin-associated glycoprotein to Rho. J. Cell Biol. 157, 565–570. 10.1083/jcb.200202010 12011108 PMC2173856

[B170] Yamashita T. Mueller B. K. Hata K. (2007). Neogenin and repulsive guidance molecule signaling in the central nervous system. Curr. Opin. Neurobiol. 17, 29–34. 10.1016/j.conb.2006.12.001 17169551

[B171] Yamashita T. Tohyama M. (2003). The p75 receptor acts as a displacement factor that releases Rho from Rho-GDI. Nat. Neurosci. 6, 461–467. 10.1038/nn1045 12692556

[B172] Yiu G. He Z. (2003). Signaling mechanisms of the myelin inhibitors of axon regeneration. Curr. Opin. Neurobiol. 13, 545–551. 10.1016/j.conb.2003.09.006 14630216

[B173] Yiu G. He Z. (2006). Glial inhibition of CNS axon regeneration. Nat. Rev. Neurosci. 7, 617–627. 10.1038/nrn1956 16858390 PMC2693386

[B174] Yu J. Z. Ding J. Ma C. G. Sun C. H. Sun Y. F. Lu C. Z.. (2010). Therapeutic potential of experimental autoimmune encephalomyelitis by Fasudil, a Rho kinase inhibitor. J. Neurosci. Res. 88, 1664–1672. 10.1002/jnr.22339 20077431

[B175] Zhang Z. Xu X. Zhang Y. Zhou J. Yu Z. He C. (2009). LINGO-1 interacts with WNK1 to regulate nogo-induced inhibition of neurite extension. J. Biol. Chem. 284, 15717–15728. 10.1074/jbc.M808751200 19363035 PMC2708869

[B176] Zheng B. Atwal J. Ho C. Case L. He X. L. Garcia K. C.. (2005). Genetic deletion of the Nogo receptor does not reduce neurite inhibition *in vitro* or promote corticospinal tract regeneration *in vivo*. Proc. Natl. Acad. Sci. U.S.A. 102, 1205–1210. 10.1073/pnas.0409026102 15647357 PMC544342

[B177] Zheng B. Ho C. Li S. Keirstead H. Steward O. Tessier-Lavigne M. (2003). Lack of enhanced spinal regeneration in Nogo-deficient mice. Neuron 38, 213–224. 10.1016/S0896-6273(03)00225-3 12718856

[B178] Zhou X. Hu X. He W. Tang X. Shi Q. Zhang Z.. (2011). Interaction between amyloid precursor protein and Nogo receptors regulates amyloid deposition. FASEB J. 25, 3146–3156. 10.1096/fj.11-184325 21670066 PMC3157691

[B179] Zhou Y. Su Y. Li B. Liu F. Ryder J. W. Wu X.. (2003). Nonsteroidal anti-inflammatory drugs can lower amyloidogenic Abeta42 by inhibiting Rho. Science 302, 1215–1217. 10.1126/science.1090154 14615541

